# Descemet's Membrane Detachment During Cataract Surgery in Lattice Corneal Dystrophy Type I: Histopathological Analysis of Posterior Corneal Involvement

**DOI:** 10.7759/cureus.81431

**Published:** 2025-03-29

**Authors:** Akifumi Matsumoto, Hideki Fukuoka, Chie Sotozono

**Affiliations:** 1 Department of Ophthalmology, Kyoto Prefectural University of Medicine, Kyoto, JPN

**Keywords:** cataract surgery, corneal erosion, descemet's membrane detachment, lattice corneal dystrophy, penetrating keratoplasty

## Abstract

Lattice corneal dystrophy Type I (LCD1) is characterized by amyloid deposition in the corneal stroma, however its impact on Descemet's membrane adhesion remains poorly understood. This study reports a rare case of Descemet's membrane detachment (DMD) during cataract surgery in a patient with LCD1 and investigates the potential role of posterior corneal amyloid deposits in this complication. A 68-year-old male presented with LCD1 was admitted for cataract surgery for the left eye. DMD appeared during the irrigation and aspiration (I/A) phase after intraocular lens (IOL) implantation. After intraoperative partial removal of DM, persistent Descemet's membrane folds and corneal stromal edema developed the day after surgery. These complications did not improve over time, necessitating penetrating keratoplasty eight months later. Following the keratoplasty, the patient's vision improved to 0.5 LogMAR with an uneventful postoperative course. Pathological examination of the excised corneal tissue demonstrated the presence of Congo red stain-positive amyloid deposits in the posterior segment of the cornea, localized between the layers of Descemet's membrane and the corneal stroma. While it is established that amyloid deposition in the anterior segment of the cornea induces alterations in epithelial adhesion, leading to corneal erosions, the effects of deposits on DM adhesion in the posterior segment are yet to be fully understood. Our case's pathological findings suggested that these deposits may contribute to DMD. Therefore, careful monitoring of DM is crucial during cataract surgical interventions in patients diagnosed with LCD1.

## Introduction

Descemet's membrane (DM), the basement membrane of the corneal endothelium, contributes to maintaining the corneal transparency along with the endothelium. The thickness of DM demonstrated an increase with advancing age [1]. On average, the membrane thickness was approximately 2 micrometers in individuals aged 10 years and increased to 10 micrometers in those aged 80 years [2]. DM serves a critical role in preserving corneal clarity. Descemet's membrane detachment (DMD) is the separation of the DM from the posterior corneal stroma, and a severe complication of intraocular surgery [1,3]. DMD has been reported in the literature to occur in various ocular surgeries, including cataract surgery, keratoplasty, iridectomy, vitrectomy, and trabeculectomy [4]. Previous literature indicates that DMD occurs in an estimated 0.5% of cataract surgical procedures [5]. Consequently, DMD can lead to significant endothelial cell loss and a cascade of complications, including corneal stromal edema, formation of epithelial bullae, and substantial visual impairment [1,6]. While DMD typically manifests as a complication of ocular surgery or trauma, its management becomes particularly challenging when it occurs in corneas with pre-existing pathology. A condition that has the potential to complicate management decisions is lattice corneal dystrophy (LCD).

LCD is a rare, hereditary ophthalmic disorder characterized by amyloid deposition within the corneal stroma, leading to a progressive deterioration of visual acuity [7]. LCD is characterized by linear opacities and amyloid deposition within the corneal stroma, forming a lattice-like pattern [8]. LCDs are categorized into distinct subtypes according to their clinical manifestations and underlying genetic variations, with Type I emerging as more prevalent [7]. It has been established that LCD1 follows an autosomal dominant inheritance pattern, characterized by the Arg124Cys (R124C) mutation in the transforming growth factor beta induced (TGFBI) gene [8,9]. Thus, LCD1 is induced by the accumulation of a mutant variant of the transforming growth factor beta induced protein (TGFBIp), encoded by the TGFBI gene, commonly referred to as kerato-epithelin [10]. These amyloid deposits manifest as linear, 'lattice-like' opacities that predominantly affect the central corneal region, whereas the peripheral cornea is frequently uninvolved [7]. The anterior localization of corneal deposits of amyloid is associated with recurrent episodes of corneal erosion and irregularities in the corneal surface topography.8 Nonetheless, the impact of amyloid deposition on the structural integrity of the cornea, beyond causing corneal epithelial erosion, remains poorly understood.

In this report, we presented a novel case report of a patient diagnosed with LCD1 who experienced DMD as an intraoperative complication during cataract surgery.

## Case presentation

**Figure 1:**
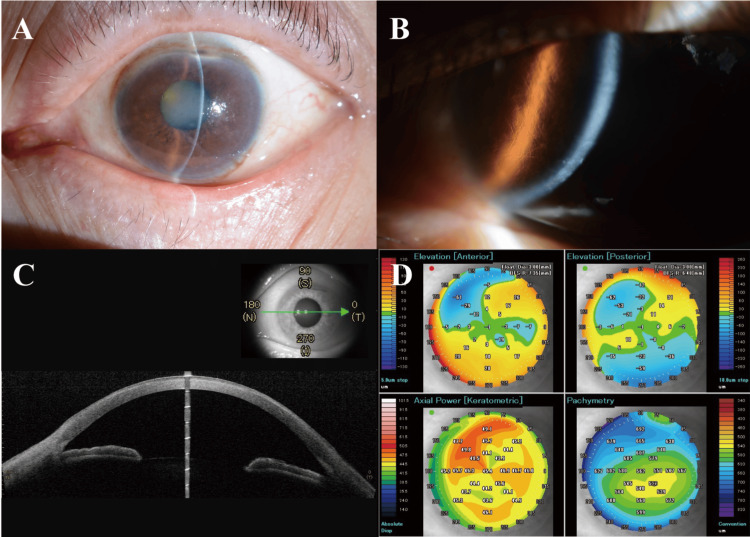
Images of the pre-operative examination before cataract surgery. (A) Slit-lamp assessment documented mild corneal opacification coexisting with significant nuclear lens sclerosis. (B) The slit-lamp image showed the amyloid deposits and 'lattice-like' opacities. (C), (D) Anterior segment optical coherence tomography (AS-OCT) images showed the anatomical structures.

A 68-year-old male patient, diagnosed with LCD1, who had previously undergone lamellar keratoplasty in the right eye at the age of 33, presented to our department with the chief complaint of progressive decreased visual acuity deterioration in left eye lasting for two years. The diagnosis of LCD1 is based on the presence of the Arg124Cys genetic variant. A preoperative examination of the left eye revealed a visually significant nuclear cataract. Furthermore, the presence of corneal opacity consistent with LCD1 was observed, as depicted in Figure 1A, 1B.

**Figure 2:**
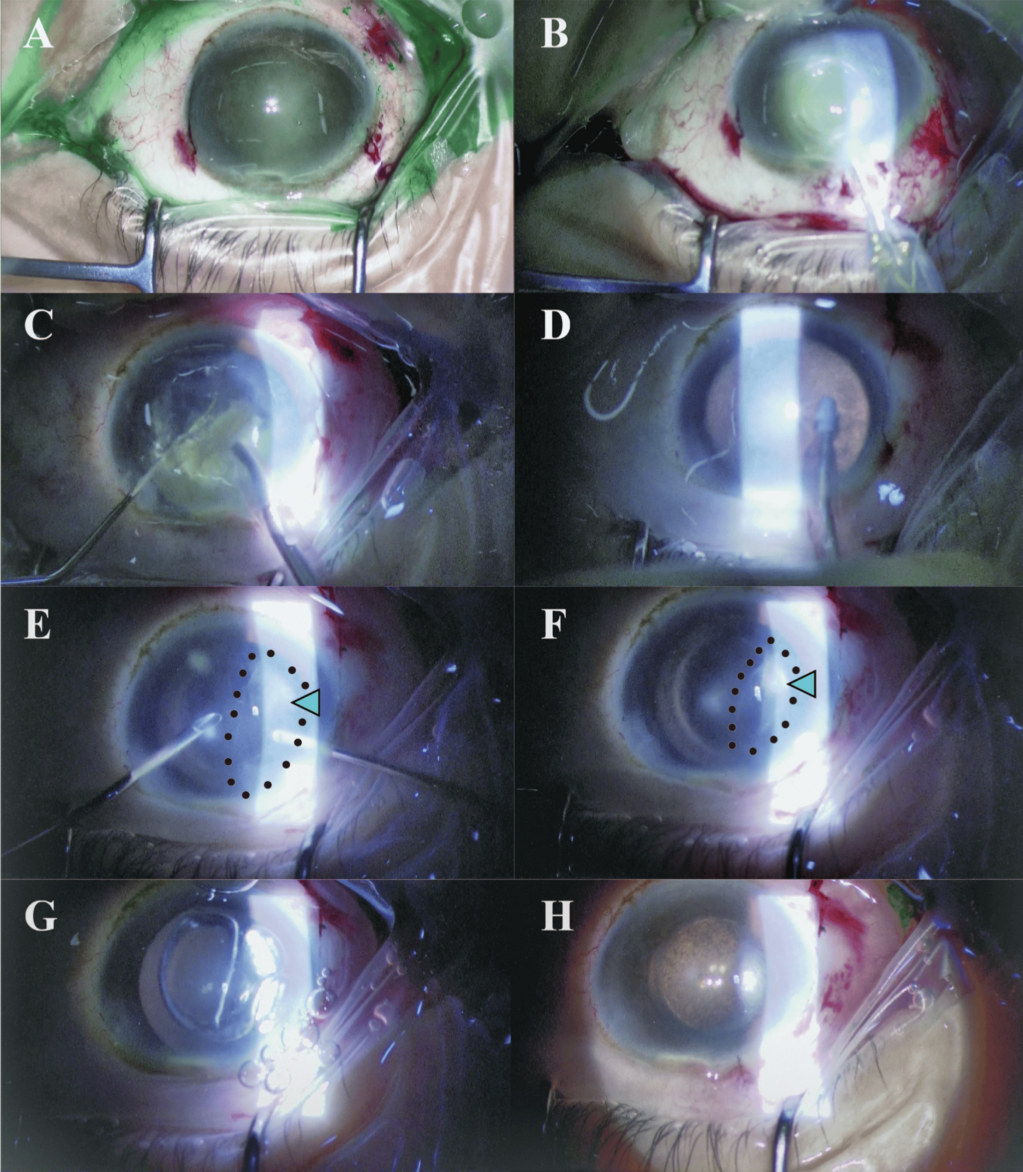
Intraoperative images of cataract surgery. (A) Intraoperative image after staining the anterior capsule with indocyanine green. (B) The capsulotomy with the Zepto nano-pulse precision capsulotomy technology. (C) Intraoperative image during phacoemulsification. (D) Intraoperative image during the irrigation and aspiration (I/A). (E), (F) After intraocular lens (IOL) implantation in the capsular bag, to remove the remaining peripheral cortical material, I/A was performed by the bimanual I/A system. Slit-lamp examination revealed extensive membranous material (green arrowhead). (G) An attempt was conducted to determine whether the folded membranous material could be unfolded using an air tamponade technique. (H) The membranous material was removed along with the elimination of the viscoelastic substance.

Preoperative examination of the left eye revealed the following measurements: The best corrected visual acuity (BCVA) was 2.0 LogMAR. Intraocular pressure (IOP) was 15.0 mmHg. Autorefractometry showed -15.75Dsph -0.50Dcyl ax. 75°. Keratometry (CASIA, Tomey Corporation, Nagoya, Japan) measured 47.1/43.7D. Axial length (IOL Master, Carl Zeiss Meditec, Dublin, CA, USA) was 23.56mm. Pachymetry (CASIA, Tomey Corporation) was 562μm (Figure 1C, 1D). Anterior segment depth (IOL Master, Carl Zeiss Meditec) was 3.50 mm. Corneal endothelial cell density was not measurable due to the corneal opacity. The patient underwent cataract extraction via indocyanine green-assisted phacoemulsification with the Zepto nano-pulse precision capsulotomy (Mynosys Cellular Devices, Inc., Fremont, CA, USA), and intraocular lens (IOL) implantation in the left eye (Figure 2A-2D).

**Figure 3:**
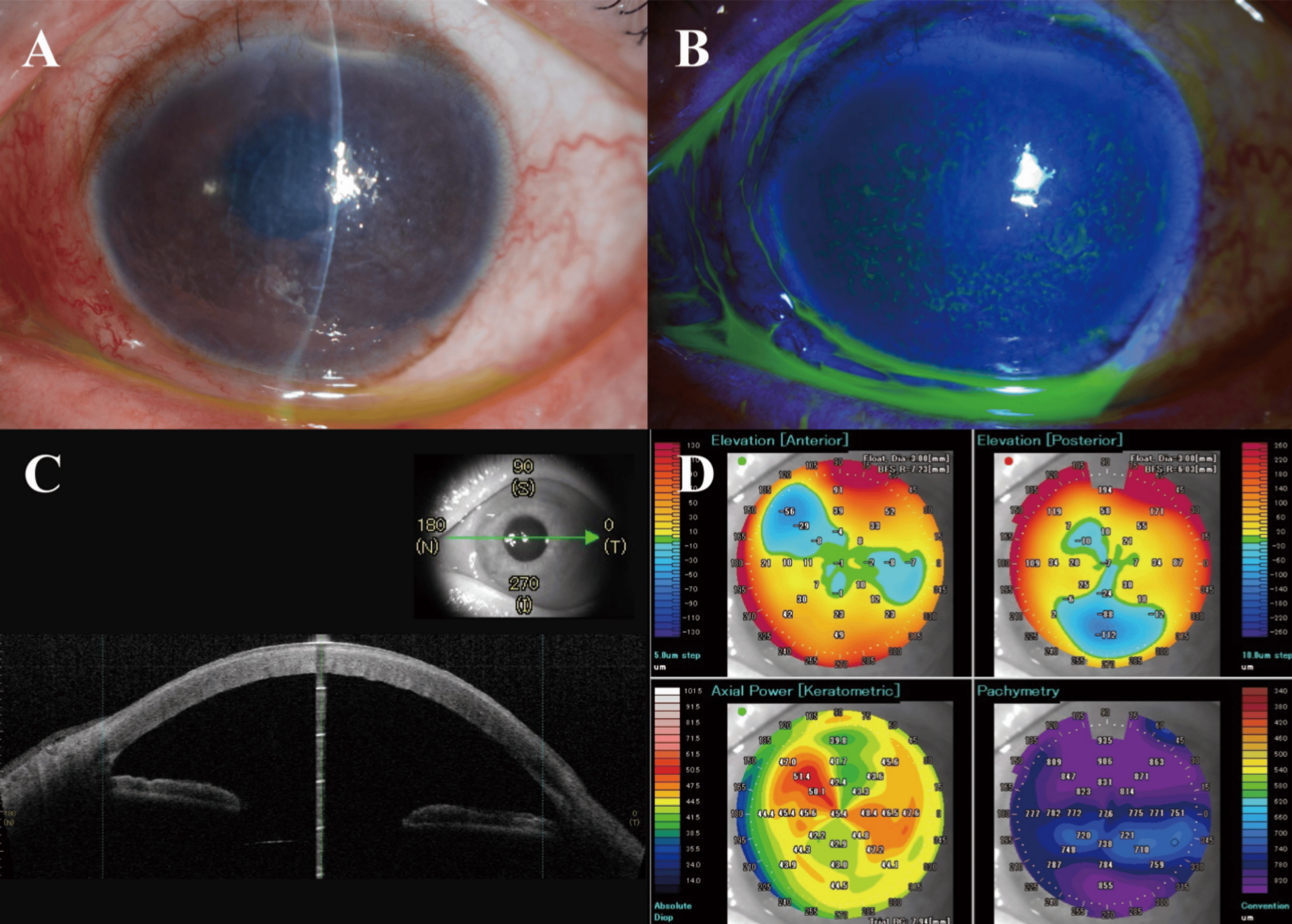
Images of the post-operative examination three weeks after cataract surgery. (A) The slit-lamp image showed mild corneal edema and Descemet's membrane folds. (B) The slit-lamp image with fluorescein showed corneal edema. (C), (D) Anterior segment optical coherence tomography (AS-OCT) images showed the anatomical structures. Significant corneal edema with increased corneal thickness is noted.

After IOL implantation, irrigation and aspiration (I/A) were performed by the bimanual I/A system to remove the remaining peripheral residual cortex (Figure 2E). During this phase, slit-lamp examination revealed extensive membranous material, the origin of which was uncertain as either the anterior capsule or Descemet's membrane. It was observed to originate from the inferior quadrant, distinctly separate from the location of the incision (Figure 2F). The folded membranous material could not be extended using an air tamponade technique, and it was removed along with the elimination of the viscoelastic substance. (Figure 2G-2H). After observing the membranous material, the surgical team suspected a Descemet's membrane detachment rather than an anterior capsule remnant, as the detached tissue extended well beyond the diameter of the previously performed continuous curvilinear capsulorhexis (CCC). A slit-lamp examination revealed corneal stromal edema with Descemet’s membrane folds the next day of the surgery. The post-operative examination after three weeks revealed persistent corneal stromal edema with Descemet’s membrane folds, and BCVA of the left eye was 1.3 LogMAR (Figure 3A-3D).

**Figure 4:**
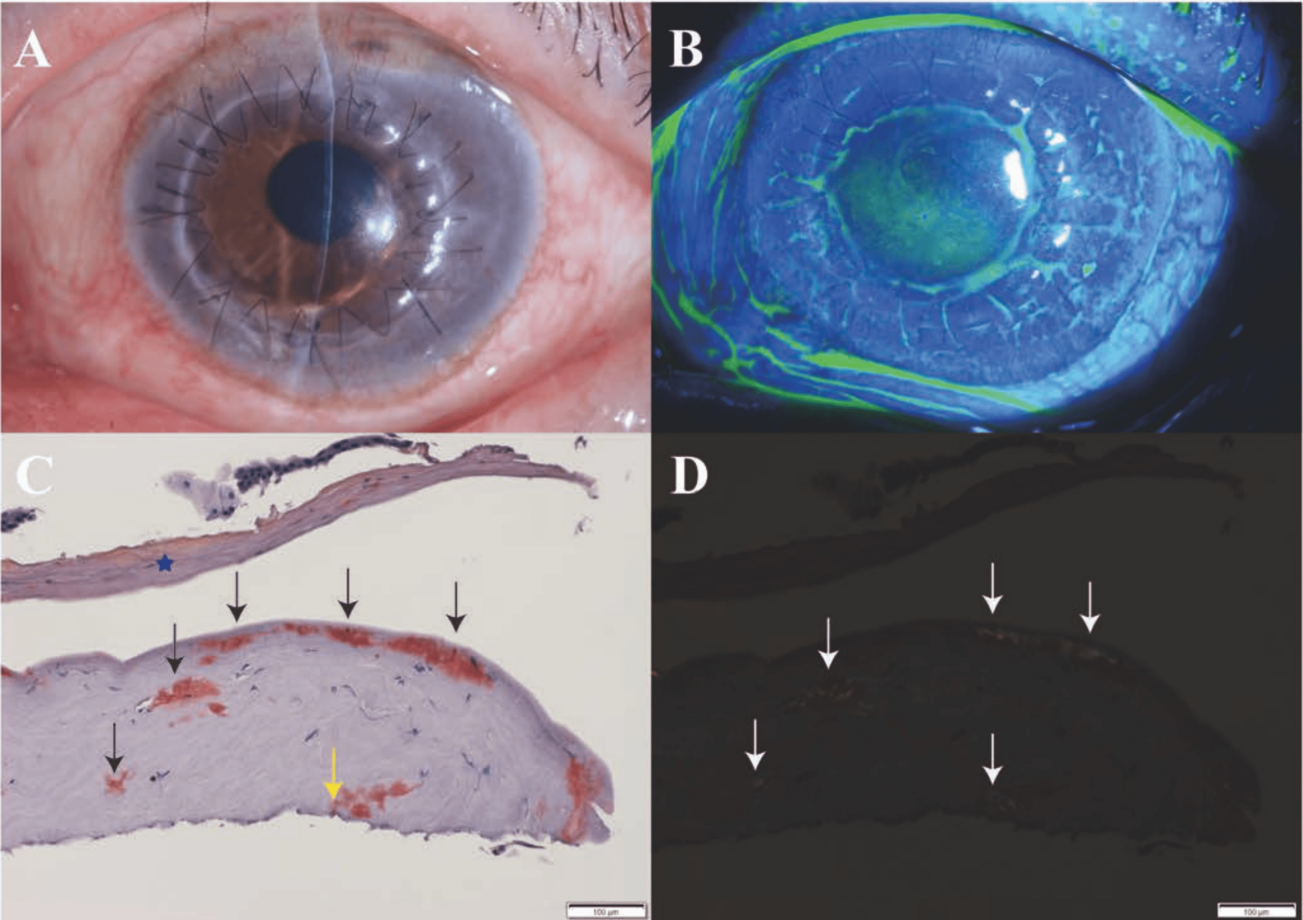
Images showingclinical examination and pathological findings results after penetrating keratoplasty. (A) The slit-lamp image of a clear grafted cornea eight weeks after penetrating keratoplasty (PKP). (B) The slit-lamp image of a clear grafted cornea with fluorescein, eight weeks after PKP. (C) The pathological examination of the full-thickness corneal specimen with Congo red stain. Congo red staining detects amyloid deposits in corneal stroma (red color, black arrow). Amyloid deposits in the posterior segment of the cornea, including Descemet's membrane area (red color, yellow arrow). The corneal epithelial layer was found to be detached (blue star) and the Descemet’s membrane was missing. The amyloid deposits extended from the stroma toward the region where Descemet's membrane was originally located. (D) Amyloid deposits were detected in the same region under polarized light (white arrow).

Although the patient's visual acuity had improved relative to preoperative levels, he expressed a desire to enhance his vision further. Eight months following cataract surgery in the left eye, penetrating keratoplasty (PKP) was performed due to LCD1 and corneal stromal opacity (Figure 4A, 4B).

Remarkably, pathological examination of the full-thickness corneal specimen, secured during the corneal transplantation procedure, disclosed amyloid deposition localized to the posterior corneal region. The amyloid deposits were projecting from the stroma toward the area where Descemet's membrane was initially present (Figure 4C, 4D). This pathological examination revealed the absence of Descemet's membrane, indicating its detachment. Subsequent post-operative evaluation conducted eight weeks after the PKP surgery revealed an uneventful postoperative course, with BCVA improving to 0.5 LogMAR.

## Discussion

The incidence of DMD attributable to incision-related factors during cataract surgery has been well-documented in previous studies [11]. We encountered a unique case wherein extensive DMD was observed during cataract surgery without any discernible trigger. Minor DMD resolves spontaneously within days after surgery. Conversely, severe DMD can manifest in compromised corneal health, culminating in corneal decompensation that necessitates transplantation [4]. Owing to compromised endothelial pump function, unaddressed extensive DMD may precipitate corneal decompensation and opacification if not timely managed. This can result in significant visual loss or a lack of improvement in vision following intraocular surgery [4,12]. In our case, during the surgery, it was not recognized that the membranous material was Descemet's membrane, and its surgical excision led to the removal of the majority of DM, resulting in extensive loss of the DM-endothelial complex. This was followed by the persistent Descemet's membrane folds and corneal stromal edema. Consequently, PKP was necessary to improve the patient's visual acuity.

In LCD1 patients, amyloid deposits initially appear in the anterior central corneal stroma as fine lattice lines, progressively extending deeper into the stroma and peripherally with age. This condition has the potential to result in visual impairment due to the development of diffuse central haze, recurrent corneal erosions (RCEs), and delayed epithelial healing following corneal surgical interventions [8,13,14]. Despite the well-documented clinical features of LCD1, to our knowledge, there have been no reports in the existing literature specifically addressing the coexistence or potential relationship between LCD1 and DMD. This lack of documented cases highlights the unique nature of our observation and underscores the importance of careful consideration when managing patients with LCD1 who undergo corneal surgical procedures.

In our case, the pathological examination of the corneal specimen, utilizing Congo red staining, revealed amyloid deposition in both the cornea's anterior and posterior segments. The amyloid deposits extended from the stroma toward the region where Descemet's membrane was originally located. Thus, we hypothesized that the presence of amyloid deposits in this region may contribute to its detachment. It has been reported that amyloid deposits in the anterior segment have been established to induce alterations in corneal epithelial adhesion, thereby precipitating corneal erosions [13]. This established mechanism in the anterior segment provides a conceptual framework for understanding potential similar effects in the posterior segment. Additionally, our pathological examination revealed detachment of the corneal epithelial layer, further supporting the notion that amyloid deposits can disrupt cellular adhesion. However, the impact of amyloid deposition in the posterior segment remains to be elucidated. Furthermore, we propose that amyloid deposition in the posterior segment similarly influences cell-matrix adhesion molecules and basement membrane components, as observed in the anterior segment. This alteration may predispose the tissue to DMD.

## Conclusions

Our case demonstrates that amyloid deposition in LCD1 may have implications beyond the anterior corneal segment. The pathological findings suggest that these deposits may contribute to Descemet's membrane detachment during intraocular surgery. To mitigate the risk of severe complications, careful monitoring for DMD is crucial during surgical interventions in patients with LCD1. Further research is needed to understand the mechanisms by which amyloid deposits affect basement membrane adhesion in different corneal layers.
